# Intravenous dexmedetomidine versus levobupivacaine for hemodynamic response towards skull pin insertion

**DOI:** 10.6026/973206300210434

**Published:** 2025-03-31

**Authors:** Aishwarya Srinivasan, Shraddha Naik, Jamale P.B

**Affiliations:** 1 Department of Anesthesiology, Krishna Institute of Medical Sciences, Krishna Vishwa Vidyapeeth (Deemed To Be University), Karad, Maharashtra, India

**Keywords:** Hemodynamic response, skull pin head, intravenous dexmedetomidine, levobupivacaine, local anesthesia, fentanyl

## Abstract

Local anesthetics such as levobupivacaine, along with fentanyl and intravenous dexmedetomidine, have shown potential in reducing sympathetic activation and tension. Therefore, it is of interest to compare intravenous dexmedetomidine with levobupivacaine. Patients were randomly assigned to two groups of 40 each, designated as Group D and Group S, with measures including baseline, heart rate, mean arterial pressure, systolic blood pressure, and diastolic blood pressure. At baseline, no significant difference in saturation was seen across the Groups (p = 0.10). Likewise, during induction and at subsequent intervals (1 minute before and after skull pin placement, as well as at 3, 5, and 10 minutes thereafter), Spo2 levels were similar across the two groups (all p > 0.05). We concluded that the scalp block using fentanyl-levobupivacaine and intravenous dexmedetomidine has comparable efficacy in diminishing the hemodynamic response associated with the skull pin head.

## Background:

The skull pin head holder is employed to ensure the stable positioning of the patient's head throughout neurosurgical interventions. The head holder, commonly referred to as a skull clamp, plays a vital role in reducing even the most minor movements during micro neurosurgery, thereby mitigating potential risks. The insertion of skull pin may elicit pain through the stimulation of nerve endings located in the scalp and periosteum, resulting in a hemodynamic response and an elevation in stress hormone levels [1, 2-3]. The utilization of a skull pin head holder for head stabilization during craniotomy elicits a significant noxious stimulus and activates the sympathetic nervous system [4, 5]. Additionally, it may increase cerebral blood flow and intracranial pressure [6]. Multiple anesthetic and pharmacological techniques have been utilized to reduce the hemodynamic response associated with the insertion of skull pin [7, 8]. A study found that, the dexmedetomidine increased perioperative hemodynamic stability in patients undergoing brain tumor surgery. Compared with fentanyl, the trachea was intubated faster without respiratory depression [9]. However, there are few comparative studies examining the effectiveness of these medicines, specifically in the setting of craniotomies. Understanding which pharmacological intervention, or combination thereof, offers the best hemodynamic stability during skull pin insertion is critical for improving patient outcomes and improving the safety profile of this neurosurgical surgery. Therefore, it is of interest to compare the efficacy of intravenous dexmedetomidine, fentanyl and 0.5% levobupivacaine scalp block in attenuating the hemodynamic response.

## Materials and Methods:

The current prospective, randomized clinical study was conducted over a period of 18months and a detailed pre-anesthetic check-up including general (electrocardiogram and chest radiogram) and systemic examination (complete blood count, blood sugar, renal function test, coagulation profile, serum electrolytes) and explanation about the anesthesia technique prior to the surgery. Followed which patients were randomly divided into two groups of 40 each *i.e.* Group D (intravenous dexmedetomidine) (4mcg/cc by taking 1amp (200mcg) diluted with 48cc of Normal saline in a 50cc syringe. Loading dose was calculated acc. to body weight of the patient in a dose of 1mcg/kg for 10 minutes. Later, maintenance dose was calculated in a dose of 0.5mcg/kg/hr) and Group S (scalp block) (25ml of 0.5% LB to which 1mcg/ml (total 25mcg) of fentanyl).

## Anesthesia technique:

Induction of anesthesia was done with standard induction protocol for all patients with IV midazolam 0.03mg/kg, propofol (2mg/kg), fentanyl (2mcg/kg) and vecuronium (0.15mg/kg). After confirming placement of ET tube by auscultation and positive end-tidal carbon dioxide graph, end-tidal Sevoflurane monitoring was established. Anesthesia was maintained with 1.2 minimum alveolar concentrations (minimum alveolar concentration). In Group D patients, after 10 minutes of loading dose, infusion rate was calculated according to weight and 0.5mcg/kg/hr, while the patient was handed over immediately after induction for insertion of Skull pins. In Group S patients, after induction, bilateral scalp block was performed using the drug made (25ml of 0.5%LB+25mcg Fentanyl) with 12.5ml of drug injected on each side, covering 6 Groups of scalp nerves. After fixing the skull pin, the total duration of our study is 10 minutes. The following parameters were measured at baseline/before induction, after induction of anaesthesia (after loading dose in group D), 1 minute before skull pin insertion (T-1) and after pinning at 1,3,5,10 minutes (T1, T3, T5, T10) for heart rate, systolic blood pressure, diastolic blood pressure and Mean Arterial Pressure.

## Inclusion criteria:

[1] Patient age between 18 to 65 years.

[2] American society of anaesthesiologist physical status grade 1 and 2.

## Exclusion criteria:

[1] Glass coma scale <9 (Severe brain injury).

[2] Preoperative heart rate <50 beats per minute.

[3] Known allergies to local anaesthetics or dexmedetomidine or fentanyl.

[4] Treatment with beta-blockers.

[5] Comorbidities like- Left ventricular dysfunction, uncontrolled hypertension, severe hepatic and renal diseases and first/second degree heart blocks.

[6] Pregnant and lactating patients

[7] Redo craniotomies

## Statistical analysis:

We have used Fisher's exact test, unpaired student's test, Univariate analysis of variance and the general linear model for repeated measures and it is considered statistically significant when the p-value is less than 0.05.

## Results:

Table 1 shows that, mean age for group D is 41.25 years ±13.930 and group S, it is 41.20 years ±14.084. The standard error of the mean is 2.202 for group D and 2.227 for group S. A t-test yielded a T value of 0.016 and a p-value of 0.98, indicating no statistically significant difference between the two groups. Table 2 shows that, in group D, there are 20 females (50.0%) and 20 males (50.0%) In group S, there are 21 females (52.5%) and 19 males (47.5%). The overall distribution shows 41 females (51.2%) and 39 males (48.8%) out of the total 80 subjects. A chi-squared value of 0.05 and a p-value of 0.82, indicates no statistically significant difference between the two groups. Table 3 shows that, group D is 159.07 cm ± 5.465 and group S, it is 158.88 cm ± 4.603. The standard error of the mean is 0.864 for group D and 0.728 for group S. A t-test resulted in a T value of 0.17 and p-value of 0.86, indicating no statistically significant difference. Table 4 shows that, mean weight for group D is 56.13 kg ± 8.847 and group S is 54.40 kg±10.025. The standard error of the mean is 1.399 for group D and 1.585 for group S. A t-test resulted in a T value of 0.81 and a p-value of 0.41, indicating no statistically significant difference. Table 5 shows that, group D, 17 subjects (42.5%) are American Society of Anesthesiologists I and 23 subjects (57.5%) are American Society of Anesthesiologists II. In group S, 19 subjects (47.5%) are American Society of Anesthesiologists I and 21 subjects (52.5%) are American Society of Anesthesiologists II. Overall, there are 36 American Society of Anesthesiologists I subjects (45.0%) and 44 American Society of Anesthesiologists II subjects (55.0%) out of the total 80 subjects. A chi-squared value of 0.20 and a p-value of 0.65, indicates no statistically significant difference. Table 6 shows that, at baseline, there was no significant difference in saturation between the groups (p = 0.10). Similarly, after induction and throughout subsequent time points (1 minute before and after skull pin insertion and at 3, 5 and 10 minutes after), Spo2 remained comparable between the two groups (all p > 0.05). Table 7 shows that, at base-line group D had a significantly higher mean blood pressure (90.60 mmHg) compared to the Standard group (83.85 mmHg) with a p-value of 0.02. However, after induction individuals in group D showed a drop in heart rates (mean-76.28bpm) and there was statistically significant difference between both groups (p-0.03). Throughout subsequent time points (1 minute before and after skull pin insertion and at 1 minute after), there were not statistically significant differences between the groups in the heart rates (p > 0.05 for all comparisons). In addition to above, at 3 and 5minutes, even though both groups showed raise in heart rates due to skull pin insertion, individuals in group D showed lower heart rates than individuals in group S and it was statistically significant( at T3, p-0.01; at T5,p-0.04). 10 minutes after skull pin insertion, heart rates between both groups were stabilized (p>0.05). Table 8 shows that, initially, baseline systolic blood pressure was similar between the groups (D: 125.25 mmHg, S: 124.40 mmHg, p = 0.80). After induction and at T-1 intervals, systolic blood pressure decreased in both groups, with significant difference observed (D: 106.18 mmHg, S: 115.73 mmHg, p = 0.028; D-105.33mmHg, S-114.60mmHg, p=0.042). At subsequent time points after skull pin insertion (T1, T3, T5, T10), systolic blood pressure showed slight variations between the groups, with group D showing lower systolic blood pressure than group S, though differences were not statistically significant (p > 0.05). Overall, the study suggests comparable effects on systolic blood pressure between group D and group S treatments across the measured time intervals. Table 9 shows that, at baseline, there was no significant difference in diastolic blood pressure between the groups (p = 0.1). After Induction, diastolic blood pressure was significantly lower in the group D individuals (70.40 ± 7.417) compared to the group S individuals (76.00 ± 8.803), with a p-value of 0.003. One minute before skull pin insertion (T-1), diastolic blood pressure was also significantly lower in the group D individuals (72.93 ± 8.401) compared to the group S individuals (77.65 ± 10.633), with a p-value of 0.03. However, at subsequent time points (1 minute after and at 3, 5 and 10 minutes after skull pin insertion), there were no significant differences (all p > 0.05). Table 10 shows that, at baseline, there was no significant difference in Mean Arterial Pressure between the groups (p = 0.17). After Induction and at one minute before skull pin insertion (T-1), Mean Arterial Pressure was lower in the group D (77.28 ± 9.175) compared to group S (82.47 ± 14.888), significantly (p = 0.021; p=0.043 respectively). At 1 minute after skull pin insertion, there was no significant differences in Mean Arterial Pressure between the groups (all p > 0.05). However, at 3,5,10 minutes after skull pin insertion, the group D individuals showed significantly lower Mean Arterial Pressure (81.47 ±9.717;84.20 ±4.717;85.20 ± 4.121) compared to the individuals in group S (89.03 ±11.417; 90.10 ±7.653; 90.40 ± 5.518), with a p-value of 0.047 respectively. Table 11 shows that, Bradycardia only was present in 2.5% (1 out of 40) of the individuals of group D, while 5% (2/40) had only Bradycardia in group S. Bradycardia combined with hypotension occurred in 37.5%(15 out of 40) of individuals in group D and none showed in group S(0%). While Hypotension alone was observed in 55.0% (22 out of 40) of the individuals in Group D conversely, only 27.5% (11 out of 40) of the Standard group experienced Hypotension without Bradycardia. A majority (67.5%) of individuals in group S experienced no adverse effects, compared to 37.0% in group D. The Chi-square test indicates a significant difference between the groups (χ^2^ = 42.05, p < 0.05), suggesting that scalp block is associated with a lower incidence of adverse effects, particularly Bradycardia and Hypotension, compared to the intravenous dexmedetomidine treatment. None of the patients in either group warranted the use of Inj. Mephentermine boluses. Bradycardia and Hypotension was settled by reducing Sevoflurane and by increasing fluid rate. Table 12 shows that, the majority of subjects did not require rescue medication: 85.0 %( 34 out of 40) in group D and 92.5 %( 37 out of 40) in group S. A small percentage required rescue doses at different time points: 5.0%(2 out of 40) at T10 and 10.0%(4 out of 40) at T5 in group D, compared to 2.5%(1 out of 40) at T10 and 5.0%(2 out of 40) at T5 in group S. The Chi-square test shows no significant difference between the groups (χ^2^ = 1.13, p = 0.57), indicating similar rates of needing rescue medication between intravenous dexmedetomidine and the scalp block treatment in this study.

## Discussion:

In our study, age, gender, mean heights and weights and American Society of Anesthesiologists status among the two groups were comparable and did not influence the nature of the study in any way. The comparable data observed between groups in our study suggest that demographic parameter and American Society of Anesthesiologists status is unlikely to be a confounding factor influencing the effectiveness or safety of anesthesia methods. This finding were consistent with earlier research by Bala *et al.* in 2022, which reported that participants in Group R had a mean age of 41.10 years ± 12.99, whereas those in Group D had a mean age of 37.61 years ± 13.24, with a p value greater than 0.05. The proximity in age ranges between the two groups indicates that age-related biases are improbable, thereby bolstering the validity of the results [10]. In a similar manner, the study conducted by Thongrong *et al.* in 2017 also ensured an equal distribution of gender across the groups, comprising 7 males and 23 females in each group. This balance guarantees that any detected variations in method preferences or outcomes can be more accurately ascribed to the treatments themselves, rather than to gender bias [11]. In Bala *et al.* 2022 study reported that participants in Group R had an average height of 158.56 cm ± 5.35 cm, whereas those in Group D averaged 157.48 cm ± 6.19 cm. Despite the slight numerical differences in mean heights between the groups, the calculated p-value of 0.264 indicates no statistically significant difference in height distribution, suggesting that height was balanced across the anaesthesia method groups in Bala's study [10]. A study found that, dexmedetomidine inhibits steroid biosynthesis only in high concentrations. In the concentrations designed to provide either acute anesthesia or chronic sedation, dexmedetomidine does not cause the potent inhibitory effect on steroid genesis seen after etomidate use. As this imidazole α2-adrenergic agonist is highly efficacious as a sedative/hypnotic agent in the low nanomolar range, an important biologic effect on steroid genesis probably will not occur clinically [12]. These findings collectively suggest that both group D and group S maintained comparable SPO2 levels throughout procedural stages, indicating their effectiveness in maintaining adequate oxygenation. The transient difference observed in Akshaya's study immediately after pinning highlights potential short-term effects that may not necessarily impact overall oxygenation stability over time. Overall SPO2 levels remained comparable and within clinically acceptable ranges for both anaesthesia methods in the current study and throughout various procedural stages. Sahana *et al.* in 2021 study, heart rates showed no statistically significant differences throughout time intervals after skull pin insertion, with p vale>0.05 at all times. Individuals in intravenous dexmedetomidine group had higher baseline heart rate (81.1 ±13.4) than individuals in control group (77.9 ±12.7). Later in the study, pair wise comparison revealed a significant increase in heart rate, at 1 and 3 min after skull pin insertion in both the groups(control group-84.9 ±14.8,intravenous dexmedetomidine group-87.2 ±17.0) with p values being>0.05 at both times. However at the end of 15 minutes both groups maintained comparable heart rate [13]. In our study, before induction, there was no significant difference in systolic blood pressure between the groups (D: 125.25 mmHg, S: 124.40 mmHg, p = 0.80). However, after induction and at T-1 intervals, systolic blood pressure decreased in both groups, with significant difference observed (D: 106.18 mmHg, S: 115.73 mmHg, p = 0.028; D-105.33mmHg, S-114.60mmHg, p=0.042). At subsequent time points after skull pin insertion (T1, T3, T5, T10), systolic blood pressure showed slight variations between the groups, with group D showing lower systolic blood pressure than group S, though differences were not statistically significant (p > 0.05). Overall, the study suggests comparable effects on systolic blood pressure between Dexmedetomidine and Scalp block across the measured time intervals. Most of the patients in group D exhibited lower systolic blood pressure after induction but was not significant enough to require intervention. 22 patients in this group showed significant hypotension (*i.e* >15% fall in baseline) but did not require any intervention. This indicates that the group D was also significantly effective in attenuating pressor response to skull pin insertion across all measured intervals compared to group S. Overall, these studies highlight the importance of selecting anaesthesia methods based on their specific effects on systolic blood pressure and overall hemodynamic stability. While intravenous dexmedetomidine appears to offer consistent or potentially advantageous control over systolic blood pressure as compared to Scalp block group, in some contexts, such as maintaining lower and more stable systolic blood pressure readings, the clinical implications and optimal choice of anaesthesia should consider individual patient characteristics, procedural requirements and the overall balance between hemodynamic control and patient safety. In the study conducted by Singh *et al.* in 2021, diastolic blood pressure recordings in the groups D (74.29 ± 12.11) and S (75.97 ± 16.91) at BL were comparable. After Induction, there was a fall in diastolic blood pressure in both the groups but not significant. However, later at 1, 2 and minutes after pinning, diastolic blood pressure showed a rising trend in Group D than group S with P value<0.05 at each of these intervals. While, eventually diastolic blood pressure stabilized to baseline levels in group D, group S showed a lower stable diastolic blood pressure throughout these time intervals without fluctuations [14]. Overall, while the current study and referenced literature indicate varying impacts on diastolic blood pressure across anaesthesia methods, intravenous dexmedetomidine consistently shows a tendency towards lower diastolic blood pressure levels compared to scalp block-based regimens during certain procedural phases. These findings emphasize the importance of selecting anaesthesia protocols tailored to individual patient needs, procedural requirements and the desired hemodynamic goals. Bala *et al.* in 2022 study, while Mean Arterial Pressure values were statistically similar between the two groups at all-time points except T10-T20 where values were statistically significant. Though the attenuation of Mean Arterial Pressure in response to pin insertion was maintained throughout, there was more than 20% decrease in Mean Arterial Pressure values from T5-T20 as compared with baseline in both the groups. But this decrease was more in group D (26-29% from baseline) than group R (21-22%) leading to statistical significant difference at T10 to T20 between the two groups (p < 0.020). This can be attributed to hypotension caused by intravenous dexmedetomidine [10]. Overall, while the current study and previous studies' findings indicate no significant Mean Arterial Pressure differences between the two groups, variability across studies underscores the influence of anaesthesia choice on cardiovascular parameters during procedures involving skull pin insertion. Intravenous dexmedetomidine potential to lower Mean Arterial Pressure may offer benefits in certain contexts, such as reducing intraoperative bleeding and optimizing surgical conditions, but careful titration is essential to mitigate risks of excessive Hypotension. In the current study, individuals in Group D showed more adverse effects than individuals in group S. With hypotension (fall in Bp >15% of baseline) seen in 22 out of 40 individuals(55%), while only half the number showed hypotension in Group S(27.5%) and this was statistically significant with p<0.05. Some individuals also showed bradycardia (fall in Heart rate>15% of baseline) more in Group D than Group S (16:2). These effects were mostly seen in Group D after induction and generally at 1 minute before skull pin insertion. Although after pin insertion there was rise in heart rate and blood pressure in both the groups, group D still had lower heart rates or blood pressure than baseline rates as compared to group S individuals. Hence, none of the groups warranted use of any management for these effects. In a study, author found that, dexmedetomidine administered for a scalp block effectively reduced hemodynamic responses associated with skull pin insertion in patients undergoing craniotomy under general anesthesia [15].

## Aim:

To assess the efficacy of intravenous dexmedetomidine and scalp block with Fentanyl along with 0.5% LB to attenuate the Hemodynamic response to skull pin insertion in craniotomies after induction with routine general anesthesia.

## Conclusion:

The scalp block utilizing fentanyl-levobupivacaine and intravenous dexmedetomidine demonstrates comparable efficacy in mitigating the hemodynamic response associated with skull pin insertion. However, the scalp block provides superior analgesia. In contrast, while intravenous dexmedetomidine is generally well-tolerated; it necessitates careful consideration due to potential adverse effects.

## Figures and Tables

**Table 1:** Age distribution

	**Group**	**N**	**Mean**	**Std. Deviation**	**Std. Error Mean**	**P Value**
Age	D	40	41.25	13.93	2.202	T value- 0.016, p value- 0.98, non-significant
	S	40	41.2	14.084	2.227	

**Table 2:** Sex distribution

		**Count**	**Group**		**Total**
			**D**	**S**	
Sex	F		20	21	41
		% within Group	50.00%	52.50%	51.20%
	M	Count	20	19	39
		% within Group	50.00%	47.50%	48.80%
Total		Count	40	40	80
		% within Group	100.00%	100.00%	100.00%
Chi-sq value- 0.05, p value- 0.82					

**Table 3:** Height distribution

	**Group**	**N**	**Mean**	**Std. Deviation**	**Std. Error Mean**	**P Value**
Height	D	40	159.07	5.465	0.864	T value- 0.17, p value- 0.86, non-significant
	S	40	158.88	4.603	0.728	

**Table 4:** Weight distribution

	**Group**	**N**	**Mean**	**Std. Deviation**	**Std. Error Mean**	**P Value**
Weight	D	40	56.13	8.847	1.399	T value- 0.81, p value- 0.41, non-significant
	S	40	54.4	10.025	1.585

**Table 5:** American Society of Anesthesiologists status

			**Group**		**Total**
			**D**	**S**	
ASA status	I	Count	17	19	36
		% within Group	42.50%	47.50%	45.00%
	II	Count	23	21	44
		% within Group	57.50%	52.50%	55.00%
Total		Count	40	40	80
		% within Group	100.00%	100.00%	100.00%
Chi-sq value- 0.20, p value- 0.65, non-significant					

**Table 6:** Mean comparison of spo2 at different time points

	**Group**	**N**	**Mean**	**Std. Deviation**	**Std. Error Mean**	**P Value**
Baseline(BI)	D	40	98.5	1.132	0.179	0.1
	S	40	98.08	1.163	0.184	
After Induction (AI)	D	40	98.4	1.057	0.167	0.67
	S	40	98.5	1.038	0.164	
(T-1)1 min before skull pins insertion	D	40	98.85	1.122	0.177	0.37
	S	40	98.63	1.148	0.181	
(T1)1 min after skull pins	D	40	98.65	1.099	0.174	0.19
	S	40	98.33	1.141	0.18	
3 min after skull pins(T3)	D	40	98.15	1.051	0.166	0.22
	S	40	98.45	1.154	0.182	
5 min after skull pins(T5)	D	40	98.53	1.154	0.183	0.11
	S	40	98.13	1.09	0.172	
10 min after skull pins(T10)	D	40	98.53	1.062	0.168	0.74
	S	40	98.45	1.011	0.16	

**Table 7:** Heart rate

	**Group**	**N**	**Mean**	**Std. Deviation**	**Std. Error Mean**	**P Value**
Baseline	D	40	90.6	11.261	1.781	0.02
	S	40	83.85	14.08	2.226	
After Induction (AI)	D	40	76.28	8.64	1.84	0.03
	S	40	82.6	12.791	1.706	
(T-1)1 min before skull pins insertion insertion	D	40	79.08	10.184	1.61	0.61
	S	40	82.3	11.078	1.752	
(T1)1 min after skull pins	D	40	86.28	10.105	1.598	0.51
	S	40	84.7	10.922	1.727	
(T3)3 mins after skull pins	D	40	92.15	10.475	1.656	0.01
	S	40	88.38	8.587	1.674	
(T5)5 mins after skull pins	D	40	85.1	11.675	1.846	0.04
	S	40	91.78	8.401	1.803	
(T10)10 mins after skull pins	D	40	89.83	12.59	1.991	0.35
	S	40	91.35	11.251	1.779	

**Table 8:** Systolic blood pressure

	**Group**	**N**	**Mean**	**Std. Deviation**	**Std. Error Mean**	**P Value**
Baseline	D	40	125.25	15.142	2.394	0.8
	S	40	124.4	15.219	2.406	
After Induction (AI)	D	40	106.18	12.43	1.965	0.028
	S	40	115.73	8.155	1.289	
(T-1)1 min before skull pins insertion	D	40	105.33	12.97	2.051	0.042
	S	40	114.6	7.665	1.212	
(T1)1 min after skull pins	D	40	114.13	12.527	1.981	0.16
	S	40	117.33	6.9	1.091	
3 min after skull pins	D	40	119.65	11.902	1.882	0.17
	S	40	122.65	6.573	1.039	
5 min after skull pins	D	40	124.08	11.911	1.883	0.2
	S	40	126.85	6.845	1.082	
10 min after skull pins	D	40	123.6	10.782	1.705	0.08
	S	40	127.35	7.957	1.258	

**Table 9:** Diastolic blood pressure

	**Group**	**N**	**Mean**	**Std. Deviation**	**Std. Error Mean**	**P Value**
Baseline	D	40	75.15	11.731	1.855	0.1
	S	40	71.6	6.808	1.076	
After Induction (AI)	D	40	70.4	7.417	1.173	0.003
	S	40	76	8.803	1.392	
(T-1)1 min before skull pins insertion	D	40	72.93	8.401	1.328	0.03
	S	40	77.65	10.633	1.681	
(T1)1 min after skull pins	D	40	72.28	7.693	1.216	0.09
	S	40	75.88	10.964	1.734	
3 min after skull pins	D	40	71.7	6.779	1.072	0.29
	S	40	73.88	10.999	1.739	
5 min after skull pins	D	40	72.6	6.412	1.014	0.17
	S	40	75.13	9.595	1.517	
10 min after skull pins	D	40	72.47	6.71	1.061	0.052
	S	40	75.75	7.462	1.18	

**Table 10:** Mean arterial pressure

	**Group**	**N**	**Mean**	**Std. Deviation**	**Std. Error Mean**	**P Value**
Baseline(BI)	D	40	90.38	4.216	0.667	0.17
	S	40	89.23	3.214	0.508	
After Induction (AI)	D	40	77.28	9.175	2.241	0.021
	S	40	82.47	14.888	2.354	
(T-1)1 min before skull pins insertion	D	40	74.1	4.235	0.67	0.043
	S	40	84.3	9.91	0.776	
(T1)1 min after skull pins	D	39	84.56	4.844	0.776	0.37
	S	40	88.45	6.156	0.973	
3 min after skull pins(T3)	D	40	81.47	9.717	2.011	0.036
						
	S	40	89.03	11.417	1.805	
5 min after skull pins(T5)	D	40	84.2	4.717	0.746	0.02
	S	40	90.1	7.653	0.736	
10 min after skull pins(T10)	D	40	85.2	4.121	0.652	0.047
	S	40	90.4	5.518	0.873	

**Table 11:** Adverse effects

			**Group**		**Total**
			**D**	**S**	
Adverse effect	Bradycardia(BC)	Count	1	2	3
		% within Group	2.50%	5.00%	3.70%
	Bradycardia (BC)+ Hypotension(HT)	Count	15	0	15
		% within Group	37.50%	0.00%	18.50%
	Hypotension	Count	22	11	33
		% within Group	55.00%	27.50%	40.70%
	No	Count	2	27	30
		% within Group	5.00%	67.50%	37.00%
Total		Count	40	40	81
		% within Group	100.00%	100.00%	100.00%
Chi-sq value-42.05, p value-<0.05, significant					

**Table 12:** Need for rescue drug

			**Group**		**Total**
			**D**	**S**	
Rescue dose needed	NO	Count	34	37	71
		% within Group	85.00%	92.50%	88.80%
	YES AT T10	Count	2	1	3
		% within Group	5.00%	2.50%	3.80%
	YES AT T5	Count	4	2	6
		% within Group	10.00%	5.00%	7.50%
Total		Count	40	40	80
		% within Group	100.00%	100.00%	100.00%
Chi-square value-1.13, p value-0.57, non-significant					
